# Adherence to the MIND dietary pattern and sleep quality, sleep related outcomes and mental health in male adults: a cross-sectional study

**DOI:** 10.1186/s12888-022-03816-3

**Published:** 2022-03-05

**Authors:** Hosein Rostami, Karim Parastouei, Mohammad Samadi, Maryam Taghdir, Eslam Eskandari

**Affiliations:** 1 Health Research Center, Life Style Institute, Baqiyatallah University of Medical Sciences, Tehran, Iran; 2 Present Address: Exercise Physiology Research Center, Life Style Institute, Baqiyatallah University of Medical Sciences, Tehran, Iran

**Keywords:** MIND diet, Mental Health, Sleep quality, Insomnia, Daytime sleepiness, Cross-sectional

## Abstract

**Background:**

A large number of studies have investigated the association of the Mediterranean and DASH diets with psychological health as well as sleep related outcomes. However, only a few number of studies have examined the association of their newly proposed hybrid, Mediterranean-DASH Intervention for Neurodegenerative Delay (MIND) dietary pattern, with sleep quality and sleep related outcomes.

**Methods:**

This cross-sectional study was conducted on 400 male health professions (mean age 38.67 years). Dietary information was collected using a validated 168-item semi-quantitative food frequency questionnaire. The MIND score was computed based on major dietary components emphasized or minimized in this pattern. The higher the MIND score of a subject, the greater his adherence to the MIND pattern. Mental health was evaluated using the 21-item depression, anxiety, and stress scale (DASS-21). Pittsburgh Sleep Quality Index (PSQI) was used to assess sleep quality. Sleep-related outcomes (day-time sleepiness and insomnia) were also evaluated using standard questionnaires

**Results:**

No significant associations were found between adherence to the MIND diet score and odds of stress, anxiety and depression either in the crude or multivariable-adjusted models (*P* > 0.05). Nevertheless, participants with the greatest adherence to the MIND diet had lower odds of poor sleep quality (OR for T3 vs. T1: 0.56 (95% CI: 0.34, 0.92), P-trend = 0.023). The results remained significant after full adjustment for confounding variables (P-trend = 0.042). Participants in the highest tertile of MIND diet had a 42% lower odds of daytime sleepiness in the crude and multivariable-adjusted model (P-trend < 0.05). Although no significant association was observed between adherence to the MIND diet and severity of insomnia in the crude model (P-trend = 0.055), the multivariable-adjusted model showed that the highest adherence to the MIND diet was associated with lower odds of insomnia (OR for T3 vs. T1: 0.54 (95% CI: 0.31, 0.93), P-trend = 0.031).

**Conclusion:**

While no significant associations were found between adherence to the MIND diet and stress, anxiety and depression, greater adherence to the MIND diet were associated with lower odds of poor sleep quality and sleep-related outcomes.

## Introduction

The prevalence of mental disorders has been highlighted either in Iran [1] or worldwide [2] in the last decades. It is estimated that mental health-related disorders are accounting for approximately 7.4% of global burden diseases [2]. Research has indicated that mental health problems such as depression, anxiety, cognitive impairment, fatigue, and stress may be attributed to the poor quality and quantity of sleep and sleep disturbances [3–5]. In this regard, preventive strategies should be focused to tackle these mental health problems [6].

Lifestyle modifications including dietary components are considered as important primitive strategies in the prevention and treatment of psychological disorders [7]. In the recent past, the literature concerning the association of dietary components and mental health has gained more attention [8]. For instance, findings from a systematic review and meta-analysis of observational studies revealed that a healthy dietary pattern containing high intakes of fruits, vegetables, fish, and whole grains may be associated with a reduced depression risk, while no significant association was observed between the western dietary pattern and depression [9]. In addition, the results of a cross-sectional analysis in children and adolescents resulted in an increased risk of psychiatric distress and violent behaviors with higher intakes of junk foods [10]. In a cross-sectional study on 211 male adults, an association was found between poor dietary intakes and sleep disorders and insomnia [11].

Recently, a large number of studies have investigated the association of DASH and Mediterranean dietary patterns as healthy dietary patterns with psychological disorders. The results of a large prospective study indicated that consumption of Mediterranean dietary pattern was associated with lower risk of depressive symptoms in adults women [12]. On the other hand, higher adherence to DASH dietary pattern was inversely associated with daytime sleepiness score [13]. Recently, the combination of DASH and Mediterranean diet has been proposed as MIND diet (Mediterranean-DASH Diet Intervention for Neurodegenerative Delay) which emphasizes on dietary factors associated with psychological health [14]. Morris et al. found that higher MIND diet score is associated with slower decline in cognitive abilities and the MIND diet score is more predictive of cognitive decline than either of the DASH or Mediterranean diet score [14]. While only a limited number of observational studies have investigated the association of adherence to the MIND diet score and psychological disorders such as depression and anxiety, no study has been performed to investigate the association of adherence to the MIND diet with sleep quality and sleep-related outcomes. Thus, the present study is performed to examine the association between adherence to the MIND diet and psychological function in male Iranian adults.

## Methods

This cross-sectional study was performed among Iranian male adults working in different healthcare centers affiliated to Baqiyatallah University of Medical Science across Tehran province from September 2020 to March 2021. Using a previous study performed by Rostami et al. [15] investigating the association of adherence to the DASH style diet and insomnia, the minimum required sample size was calculated as 323, with alpha value and power set at 0.05 (2-sided) and 80%, respectively. However, to increase the power of our study we recruited 400 individuals. A total of 400 male adults were randomly selected by using cluster sampling method among several healthcare centers. Male adults without any history of chronic diseases (colitis, diabetes, cardiovascular diseases, cancers, hepatic and renal failure, adrenal diseases, thyroid and parathyroid related disorders, anorexia nervosa or bulimia) were included. Those participants who received anti-diabetic, anti-inflammatory, anti-depressant or anti-obesity drugs, vitamins and minerals supplements (for example: vitamin D or calcium and iron supplements) within the previous 6 months were also excluded. Written informed consent were taken from all the participants before running the study. The study was ethically approved by the Medical Ethics Committee of the Baqiyatallah University of Medical Science, Tehran, Iran (IR.BMSU.REC.1399.479).

### Dietary assessment

Dietary data were collected using a semi-quantitative Willet-format food frequency questionnaire (FFQ) containing 168 food items with standard serving sizes [16]. The validity and reliability of this questionnaire in an Iranian population has been published previously [17]. Through an in person interview with expert nutritionists, participants were asked to report their consumption frequency of a given serving of each food item per day, week, month, or year. Afterwards, the obtained values of consumption were converted to the daily grams of intake for each food item using the Iranian manual for household measures [18]. The daily nutrient and energy intake of participants were calculated by entering the daily grams of intake of each food item into the Nutritionist-IV software (First Databank; Hearst, San Bruno, CA, USA). This nutrient database is based on the USDA food composition table modified for Iranian foods.

### Construction of MIND diet score

The collected data by FFQ was used for constructing the MIND diet score. According to the method of MIND diet, 15 dietary components consist of 10 brain-healthy food groups (green leafy vegetables, other vegetables, nuts, berries, beans, whole grains, fish, poultry, olive oil, and wine) and 5 unhealthy food groups ( red meats and red meat products, butter and stick margarine, cheese, pastries, fast fried foods and sweets) are included in this dietary score. In the current study, wine was not included in the MIND diet due to the lack of information in the FFQs. To build the MIND diet score, subjects were initially classified into tertile categories based on the intakes of these food components. Subjects in the lowest tertile of brain-healthy food groups (green leafy vegetables, other vegetables, nuts, berries, beans, whole grains, fish and poultry intake) were scored 0, those in the middle tertile were given the score of 0.5 and those in the highest tertile were given the score of 1. In case of brain-unhealthy food groups (red meats and red meat products, butter and margarine, cheese, pastries, fast fried foods and sweets), the exact opposite of this scoring protocol was applied. The scores of all 14 food components were then added up to construct an overall MIND score ranging theoretically from 0 to 14 for each subject. The higher the MIND diet score of a subject, the greater his adherence to the MIND pattern.

### Demographic and anthropometric assessments

A standard questionnaire was used to obtain general demographic information including age, smoking status (yes/ no), marital status (single/ married), smoking status (yes/ no), education (elementary or lower/ high school/ college and university) by a face to face interview. Physical activity was obtained using International Physical Activity Questionnaire (IPAQ)-short form and expressed as metabolic equivalent (MET)-min/day [19]. The validity and reliability of the IPAQ has been investigated in Iranian population [20]. Height was measured to the nearest 0.1 cm using a wall-fixed measuring tape. Body weight was measured while participants were in light clothes and without shoes. Body mass index (BMI) was calculated by dividing weight (kg) to height (m^2^).

### Depression Anxiety Stress Scales (DASS)

Depression Anxiety and Stress Scale (DASS-21) was used to screen depression, anxiety and stress. DASS-21 is a valid and reliable questionnaire to measure status of negative well beings for general population [21]. The questionnaire contains 21 items with 3 subscales. Each subscale includes 7 questions in which each item rates on a 4-point Likert scale 0–3 measuring severity of depression, anxiety and stress. Higher scores shows severe negative emotion. The total score of each sub-class should be doubled since DASS-21 is the short version of DASS-42 [21]. All three main domains were devided into two categories (no anxiety (score ≤ 7) or some degree of anxiety (> 7); no depression ( score ≤ 9) or some degree of depression (> 9); no stress ( score ≤ 14) or some degree of stress (> 14). The validated DASS-21 questionnaire in an Iranian population was used in the current study [22].

### Sleep quality assessment

Pittsburgh Sleep Quality Index (PSQI) was used as a reliable and valid tool to assess sleep quality. This tool consists 19 questions with seven components scores: sleep quality, sleep latency, sleep duration, habitual sleep efficiency, sleep disturbances, use of sleeping medication, and daytime dysfunction. Each component score is rated from 0 to 3 (0, not in the past month; 1, less than once per week; 2, once or twice per week; and 3, three or more times per week). The total score ranges from 0 to 21, which higher scores indicating poorer sleep quality. Total scores greater than 5 indicated poor sleep quality while scores ≤ 5 were indicative of normal sleep quality. The validated version of this tool in an Iranian population done by farrahi et al. with acceptable validity and reliability was used in the current study [23].

### Assessment of daytime sleepiness

The persian validated form of Epworth Sleepiness Scale (ESS) was used for the assessment of daytime sleepiness [24]. The questionnaire includes 8 questions investigating sleepiness in eight daily situations. Each question is rated from 0 to 3 giving a total score of 0–24. The higher scores indicating the more excessive daytime sleepiness. The severity of sleepiness was considered as normal (score ≤ 10) and mild to moderate sleepiness ( score > 10). The validity and reliability of ESS questionnaire in an Iranian population has been published elsewhere [25].

### Assessment of insomnia

The Insomnia Severity Index (ISI) questionnaire was used to assess sleep insomnia. The ISI questionnaire has seven questions with a score range of 0–4 for each item which is stratified into four categories as follows: 0 (none), 1 (mild), 2 (moderate), 3 (severe) and 4 (very Severe). The total score ranges between 0 to 28 points and the ISI score > 7, is considered to have insomnia. The validated ISI questionnaire in an Iranian population was used in the current study [26].

### Statistical analysis

Baseline characteristics of participants across tertiles of the MIND diet score were expressed as means ± SDs for continuous variables and percentages for categorical variables. Subjects were classified into three categories based on tertiles of MIND diet score. The Chi-square test was used for comparison of categorical variables and the one-way analysis of variance (ANOVA) was used for comparison of continuous variables among tertiles of MIND score. Dietary intakes of study.

Participants across tertiles of the MIND score were compared using ANCOVA, adjusted for total energy intake, except for dietary energy intake. Stress, anxiety, depression, poor sleep quality, insomnia and daytime sleepiness scores were divided into two categories (no/minimal degree and some degree of disorder) regarding to scores. We used binary logistic regressions to estimate ORs and 95% CIs for the presence of mentioned outcomes across tertiles of the MIND score in crude and multivariable-adjusted models. Age, BMI, and total energy intake were controlled for in the first model. Additional adjustments were controlled for physical activity and smoking (yes/ no). Finally, further adjustment were applied for marital status (single/ married) and educational levels (elementary or lower/ high school/ college and university) in the third model. A P-value < 0.05 was considered as statistically significant. All statistical analyses were done using SPSS (SPSS Inc., version 22).

## Results

The general characteristics of 400 included participants are presented in Table **1**. Mean age and BMI of whole population was 38.67 ± 5.26 year and 25.28 ± 3.22 kg/m^2.^ Individuals with lower adherence to MIND diet were more physically active (Table 1). No significant differences were observed between tertiles of MIND score for age, BMI, marital status, education levels and smoking status (Table 1).

**Table 1:** Baseline characteristics of participants according to tertiles of MIND diet score (*n* = 400)

Variables	Tertiles of MIND diet score			*P*-value^b^
	** < 4.25 (*n* = 130)**	**4.25—6.15 (*n* = 130)**	** > 6.16—11.5 (*n* = 140)**	
**Age (years)**^**a**^	38.66 ± 5.12	38.60 ± 5.32	38.72 ± 5.38	0.983
**Body weight**	78.73 ± 9.09	78.85 ± 8.34	78.10 ± 10.76	0.779
**BMI (kg/m**^**2**^**)**	25.05 ± 2.79	25.49 ± 3.12	25.29 ± 3.68	0.543
**Physical-activity (MET-min/day)**	117.82 ± 37.65	104.93 ± 47.06	112.14 ± 43.00	0.52
**Work shift (hours/day)**	7.31 ± 1.44	7.06 ± 1.27	7.38 ± 1.25	0.113
**Marital status**				
Single	28 (21.5%)	23 (17.7%)	21 (15%)	0.381
Married	102 (78.5%)	107 (82.3%)	119 (85.0%)	
**Smoking status**				
Yes	5 (3.8%)	6 (4.6%)	6 (4.3%)	1.000
No	125 (96.2%)	124 (95.4%)	134 (95.7%)	
**Education**				
Elementary or lower	32 (24.6%)	38 (29.2%)	40 (28.6%)	0.818
High school	67 (51.5%)	68 (52.3%)	70 (50.0%)	
College and university	31 (23.8%)	24 (18.5%)	30 (21.4%)	
**Home ownership**				0.140
Owner	119 (91.5%)	109 (83.8%)	125 (89.3%)	
Tenant	11 (8.5%)	21 (16.2%)	15 (10.7%)	

^a^Data are mean ± standard deviation (*SD*)

^b^Obtained from ANOVA or chi-square test, where appropriate

Dietary intakes of study participants across tertiles of MIND score are provided in Table 2. Participants in the third tertile had higher intakes of beans, berries, dietary fiber, vegetables, white meat, whole grains as well as total calorie compared with those in the first tertile (*P* < 0.05). In addition, dietary intakes of red meats and total fat were significantly different across tertiles of MIND diet (*P* < 0.05).

**Table 2:** Dietary intakes of study participants across tertiles of MIND diet score^a^ (*n* = 400)

Variables	**Tertiles of MIND diet score**			*P*-value^*^
	**T1 (< 4.25)**	**T2 (4.25—6.15)**	**T3 (> 6.16—11.5)**	
**Food groups (gr/day)**				
Vegetables	227.97 ± 12.35	251.10 ± 12.29	296.23 ± 12.30	0.001
Beans	28.18 ± 6.08	39.12 ± 6.05	45.51 ± 6.05	0.001
Berries	19.39 ± 5.69	25.71 ± 5.66	51.10 ± 5.66	< 0.001
Nuts	28.18 ± 6.08	39.12 ± 6.05	45.51 ± 6.05	0.136
Whole grains	9.31 ± 1.69	12.53 ± 1.68	17.21 ± 1.68	0.006
Red meat and red meat products	62.64 ± 7.23	61.82 ± 7.20	89.68 ± 7.20	0.012
White meat	18.82 ± 3.15	24.96 ± 3.13	36.32 ± 3.13	0.001
sweets	165.93 ± 10.74	163.35 ± 10.70	188.44 ± 10.58	0.202
**Nutrients**				
Total energy (kcal/day)	1964.93 ± 67.38	2009.11 ± 67.38	2509.39 ± 64.93	< 0.001
Carbohydrate (gr/day)	373.75 ± 30.30	379.28 ± 30.15	412.10 ± 30.16	0.645
Protein (gr/day)	92.74 ± 10.13	95.87 ± 10.08	99.38 ± 10.08	0.903
Fat (gr/day)	90.82 ± 3.22	79.82 ± 3.21	89.66 ± 3.21	0.029
Fiber (gr/day)	31.68 ± 2.35	36.07 ± 2.34	43.94 ± 2.34	0.002
Folate (μg/day)	477.92 ± 38.41	526.44 ± 38.22	602.83 ± 38.24	0.080
Vitamin B6 (mg/day)	2.05 ± 0.205	2.29 ± 0.204	2.5 ± 0.204	0.315
Vitamin B12 (μg/day)	10.98 ± 6.75	20.71 ± 6.72	6.92 ± 6.72	0.337
Magnesium (mg/day)	377.28 ± 14.91	388.15 ± 14.84	383.63 ± 14.84	0.871

^a^Data are Mean ± standard error (*SE*)

^*^All values were adjusted for energy intake, except for dietary energy intake, using ANCOVA

Crude and multivariable-adjusted ORs (95% CIs) for stress, anxiety and depression across tertiles of MIND diet score are indicated in Table 3. No significant associations were observed between adherence to the MIND diet and stress in crude model (OR for T3 vs. T1: 0.73 (95% CI: 0.44, 1.19), P-trend = 0.206) or multivariable adjusted model (OR for T3 vs. T1: 0.75 (95% CI: 0.44, 1.26), P-trend = 0.280). The crude or multivariable adjusted model between the MIND diet and anxiety revealed no significant associations, (OR for T3 vs. T1: 0.82 (95% CI: 0.50, 1.34), P-trend = 0.425) and (OR for T3 vs. T1: 0.81 (95% CI: 0.48, 1.36), P-trend = 429), respectively. In addition, there was no significant association between MIND diet and depression in crude (OR for T3 vs. T1: 0.81 (95% CI: 0.49, 1.34), P-trend = 0.412) and fully-adjusted model (OR for T3 vs. T1: 0.76 (95% CI: 0.44, 1.49), P-trend = 0.315).

**Table 3:** Crude and multivariable-adjusted odds ratio (95% CI) of the associations between MIND-style diet and stress, anxiety and depression

	**T1 ( *n* = 130)**	**T2 (*n* = 130)**	**T3 (*n* = 140)**	P-trend
**Stress**				
Crude	**1.00**	0.90 (0.54–1.50)	0.73 (0.44–1.19)	0.206
Model I	**1.00**	0.90 (0.54–1.50)	0.75 (0.44–1.25)	0.270
Model II	**1.00**	0.96 (0.58–1.61)	0.78 (0.46–1.30)	0.346
Model III	**1.00**	0.94 (0.56–1.57)	0.75 (0.44–1.26)	0.280
**Anxiety**	**1.00**			
Crude	**1.00**	1.03 (0.62–1.72)	0.82 (0.50–1.34)	0.425
Model I	**1.00**	1.03 (0.62–1.72)	0.80 (0.48–1.35)	0.419
Model II	**1.00**	1.01 (0.60–1.70)	0.80 (0.47–1.34)	0.400
Model III	**1.00**	1.02 (0.61–1.72)	0.81 (0.48–1.36)	0.429
**Depression**	**1.00**			
Crude	**1.00**	1.10 (0.66–1.85)	0.81 (0.49–1.34)	0.412
Model I	**1.00**	1.11 (0.66–1.87)	0.78 (0.46–1.32)	0.370
Model II	**1.00**	1.11 (0.66–1.89)	0.78 (0.46–1.33)	0.374
Model III	**1.00**	1.09 (0.64–1.85)	0.76 (0.45–1.29)	0.315

Data are OR (95% CI)

Model I: adjusted for age, BMI, energy intake

Model II: Model I + smoking, physical activity

Model III: Model II + marital status, education

Table 4 indicates the crude and multivariable-adjusted ORs for sleep quality, daytime sleepiness and insomnia across tertiles of MIND diet. Using a crude model, the odds of poor sleep quality reduced significantly with increasing adherence to the MIND diet (OR for T3 vs. T1: 0.56 (95% CI: 0.34, 0.92), P-trend = 0.023). The results remained significant after full adjustment for confounding variables (OR for T3 vs. T1: 0.58 (95% CI: 0.34, 0.98), P-trend = 0.042). Participants in the highest tertile of MIND diet had a 42% lower odds of daytime sleepiness in the crude (95% CI: 0.34, 0.98; P-trend = 0.031) and multivariable adjusted model (95% CI: 0.35, 0.98; P-trend = 0.044). Although no significant association was observed between adherence to the MIND diet and severity of insomnia in the crude model (OR for T3 vs. T1: 0.60 (95% CI: 0.36, 1.01), P-trend = 0.055), the multivariable adjusted model showed that highest adherence to the MIND diet was associated with lower odds of insomnia (OR for T3 vs. T1: 0.54 (95% CI: 0.31, 0.93), P-trend = 0.031).

**Table 4:** Crude and multivariable-adjusted odds ratio (95% CI) of the associations between MIND-style diet and sleep quality, daytime sleepiness and insomnia

	**T1 ( *n* = 130)**	**T2 (*n* = 130)**	**T3 (*n* = 140)**	P-trend
**Poor sleep quality**				
Crude	**1.00**	0.78 (0.47–1.31)	0.56 (0.34–0.92)*	0.023
Model I	**1.00**	0.77 (0.46–1.30)	0.56 (0.33–0.95)*	0.032
Model II	**1.00**	0.78 (0.46–1.31)	0.56 (0.33–0.95)*	0.033
Model III	**1.00**	0.79 (0.47–1.34)	0.58 (0.34–0.98)*	0.042
**Insomnia**	**1.00**			
Crude	**1.00**	0.90 (0.55–1.49)	0.60 (0.37–0.98)*	0.039
Model I	**1.00**	0.91 (0.55–1.50)	0.56 (0.33–0.93)*	0.027
Model II	**1.00**	0.88 (0.53–1.47)	0.55 (0.33–0.92)*	0.024
Model III	**1.00**	0.88 (0.53–1.46)	0.54 (0.32–0.91)*	0.022
**Daytime sleepiness**	**1.00**			
Crude	**1.00**	0.63 (0.38–1.05)	0.58 (0.35–0.94)*	0.031
Model I	**1.00**	0.64 (0.39–1.06)	0.60 (0.36–1.01)	0.055
Model II	**1.00**	0.63 (0.38–1.05)	0.60 (0.36–1.00)	0.053
Model III	**1.00**	0.62 (0.37–1.03)	0.58 (0.35–0.98)*	0.044

Data are OR (95% CI)

Model I: adjusted for age, BMI, energy intake

Model II: Model I + smoking, physical activity

Model III: Model II + marital status, education

^*^P-value < 0.05

## Discussion

To the best of our knowledge, the current study is the first report investigating the association between adherence to the MIND dietary pattern and sleep quality as well as sleep outcomes including insomnia and daytime sleepiness among adults. As stated in the methods section, our study was performed among Iranian male adults working in different healthcare centers affiliated to Baqiyatallah University of Medical Science, which is a military dependent organization in Iran. Thus, the majority of personnel were men. The association of dietary patterns and sleep quality and sleep related outcomes are of great importance in those who are working in military service or military related organizations due to the fact that they are at higher risk of sleep related disorders [27, 28]. Thus, we tried to address this associations in this group of individuals in the current study. Our results indicated that greater adherence to the MIND dietary pattern was associated with better sleep quality. In addition, an inverse association was found between adherence to the MIND diet and severity of insomnia and daytime sleepiness.

Dietary modifications may have a practical role in not only improving sleep quality but also in preventing sleep disorders. The findings of a cross-sectional study on 410 female students in Iran found a significant association between short sleep duration and low quality of diet [29]. Another study on 896 American women revealed no significant relationship between short sleep duration and diet quality, while significant associations were observed between long sleep duration and lower diet quality, specially lower fruit intakes and higher consumption of empty calories [30].

As reported in our results, higher intake of vegetables, beans, berries and whole grains were observed in subjects with higher adherence to the MIND diet and higher adherence to the MIND diet was associated with better sleep quality and less sleep disorders including insomnia and daytime sleepiness. In line with our findings, results of previous studies have investigated that higher intake of specific dietary components (fibers, minerals and vitamins) are associated with better sleep duration and quality and less sleep disorders [31, 32]. The results of a cross-sectional study on 1936 Italian individuals revealed that adherence to a low inflammatory index diet (such as DASH or MIND diet) was associated with improved sleep quality [33]. Pahlavani et al. investigated the correlation between adherence to the DASH dietary pattern and daytime sleepiness score in 535 adolescent girls in Iran; the results of the their study shed light on the inverse association between adherence to DASH diet and daytime sleepiness score [13]. Another similar study on 488 adolescent girls in Iran revealed that high adherence to the DASH-style diet was associated with a lower odds of insomnia in comparison to subjects with the lowest adherence [15].

Evidence has indicated that quality of sleep, sleep duration and sleep disturbance are associated with systemic inflammation [34]. The mechanisms that might explain the associations between sleep disturbance and inflammation are not fully explored. Sleep affects two primary effector systems, the sympathetic nervous system (SNS) and the hypothalamus–pituitary–adrenal (HPA) axis, which both together shift the basal gene expression profile toward increased pro-inflammatory profile [35, 36]. Activation of β-adrenergic signaling stimulates NF-κB pathway, inflammatory gene expression, release of proinflammatory cytokines, and markers of systemic inflammation [35]. Since normal nocturnal sleep is associated with a reduction in sympathetic outflow [37], the sympathetic effector pathway activation might be considered as one of the potential mechanisms to explain the associations between sleep disturbance, short sleep duration, and increased systemtic inflammation. On the other hand, MIND dietary pattern as a hybrid of Mediterranean and DASH dietary patterns contains a variety of anti-inflmmatory components including: omega-3 fatty acids in fish and walnuts, folate in grean leafy vegetables, vitamin E in nuts and olive oil, and flavonoids in berries which may exert greater anti-inflammatory effects with regard to the mentioned dietary patterns independently. Therefore, the anti-inflammatory effects of MIND dietary pattern may justify the favorable effects on sleep quality and daytime sleepiness and insomnia. In addition, evidence has indicated that sleep disturbance may accumulate free radicals in the cerebral tissues which subsequently leads to cerebral oxidative stress [38]. It has also proven that MIND dietary pattern has antioxidant effects which support its preventive effects on cognitive impairment and sleep disorders [39]. Given the role of oxidative stress in the pathogenesis of psychological and sleep-related disorders [40, 41], the antioxidant and anti-inflammatory properties of MIND diet might protect brain health. MIND diet characterized by a limited amounts of unhealthy foods including red meats, butter, fast foods, pastries and sweets which are harmful for brain health, while it is rich in fruits and vegetables as rich sources of vitamins, minerals, flavonoids and antioxidants [14].

As reported in our results, no significant associations were found between adherence to the MIND dietary pattern and depression, anxiety and stress in the current study. Although a considerable number of studies have performed to investigate the association between adherence to the DASH and Mediterranean dietary patterns and depression, anxiety and stress, only a limited number of studies have investigated the association between MIND dietary pattern and mental health. For instance, the results of a cross-sectional study on 3176 adults found an inverse association between greater adherence to the MIND diet and odds of depression and psychological distress, while similar to our findings, no significant association was reported between adherence to the MIND diet and odds of anxiety [42]. Faghih et al. performed a cross-sectional study on 240 university students and concluded that subjects in the highest tertile of DASH score had lower means score for depression, anxiety and stress compared to those in the lowest tertile [43]. The results of another study on 580 adolescent girls revealed significant inverse association between greater adherence to the DASH diet and lower odds of depression, however, found no significant relationship between a DASH-style diet and aggression [6]. Furthermore, findings from Sadeghi et al. study on 3172 adults aged 18–55 years found that participants with the highest adherence to the Mediterranean diet had lower odds for anxiety, depression, and psychological distress compred to subjects in the lowest adherence [44].

This study has several strengths. To our best knowledge, this is the first study investigating the linkage between MIND diet score and sleep quality and sleep-related outcomes. It is also among the first studies which have investigated the association between MIND dietary pattern and depression, anxiety and stress. The statistical analysis were controlled for several potential confounders. In addition, dietary intakes were obtained using a validated and reliable FFQ. On the other hand, the current study has some limitations that should be considered while interpreting the findings. The current study can not prove the causality and the temporality of relations due to the nature of study design (cross-sectional study). Our findings can be generalizable only to a small group of populations, males health professions, and might not be generalizable to females or other populations. Moreover, study outcomes were assessed using self-reported questionnaires and are susceptible to recall bias and misreporting.

In conclusion, no significant associations were found between adherence to the MIND diet and odds of stress, anxiety and depression in this cross-sectional study on male adults. Nevertheless, our findings indicate that greater adherence to the MIND diet were associated with lower odds of poor sleep quality, daytime sleepiness and insomnia. Given the limitations of the current study, further investigations of sufficient methodological quality are needed to confirm these findings.

## Data Availability

The data used and analyzed during the current study are available from the corresponding author on reasonable request.
